# Production of Alkaline Cellulase by Fungi Isolated from an Undisturbed Rain Forest of Peru

**DOI:** 10.1155/2012/934325

**Published:** 2012-11-19

**Authors:** Karin Vega, Gretty K. Villena, Victor H. Sarmiento, Yvette Ludeña, Nadia Vera, Marcel Gutiérrez-Correa

**Affiliations:** Laboratorio de Micología y Biotecnología, Universidad Nacional Agraria La Molina, Avenida La Molina s/n, Lima 12, Peru

## Abstract

Alkaline cellulase producing fungi were isolated from soils of an undisturbed rain forest of Peru. The soil dilution plate method was used for the enumeration and isolation of fast growing cellulolytic fungi on an enriched selective medium. Eleven out of 50 different morphological colonies were finally selected by using the plate clearing assay with CMC as substrate at different pH values. All 11 strains produced cellulases in liquid culture with activities at alkaline pH values without an apparent decrease of them indicating that they are true alkaline cellulase producers. *Aspergillus* sp. LM-HP32, *Penicillium* sp. LM-HP33, and *Penicillium* sp. LM-HP37 were the best producers of FP cellulase (>3 U mL^−1^) with higher specific productivities (>30 U g^−1^ h^−1^). Three strains have been found suitable for developing processes for alkaline cellulase production. Soils from Amazonian rain forests are good sources of industrial fungi with particular characteristics. The results of the present study are of commercial and biological interest. Alkaline cellulases may be used in the polishing and washing of denim processing of the textile industry.

## 1. Introduction

Plant biomass is one of the most abundant renewable resources for many purposes, and it is mainly composed of three types of polymers: cellulose, hemicellulose, and lignin that are strongly intermeshed and chemically bonded by noncovalent forces and by covalent cross-linkages. The rigid and complex molecular polymeric structure of cellulosic biomass makes lignocellulose highly resistant to chemical attack, solubilisation, and bioconversion. Physical or chemical pretreatment procedures which break down the lignocellulosic structures and thereby enhance the enzymatic accessibility are required for the conversion of biomass into several possible bioproducts [1, 2]. The enzymatic hydrolysis of cellulose materials involves synergistic actions of cellulases as well as xylanases and other enzymes [3, 4]. Cellulases are relatively costly enzymes, and a significant reduction in cost will be important for their commercial use. Most industrial cellulases are produced by fungi in submerged fermentation. *Trichoderma reesei* is the most important fungal species used for cellulase production although it produces low levels of *β*-glucosidase [5]. Some *Aspergillus *species are also important cellulase producers with higher levels of *β*-glucosidase than *T. reesei* [6].

The use of enzymes for processing cotton fiber in replacement of chemical and physical methods such as the use of alkali and washing with stones is relatively recent and has proven to be more effective because it produces less wear on the fabric; it is cheaper and has a reduced environmental impact [7]. In the enzymatic cleaning, the use of pectinases, cellulases, and proteases has been evaluated and found a synergistic effect between them. The mixture of cellulases and pectinases is the most effective [8]. The enzymatic cleaning is carried out at neutral pH [7], and, although there are pectinases that work optimally at this pH, most commercial cellulases have optimal activity at acidic pH. Therefore, sequential treatment was done by adjusting the pH fiber at each stage [8]. Cellulases have recently been found with optimal activity at neutral to alkaline pH, and these are gradually being incorporated into fiber treatment processes, especially in the polishing and washing of denim processing because the alkaline or neutral pH reduces textile backstaining [9–11]. Furthermore, it was observed that neutral cellulases are less aggressive wear cotton fibers and are preferred as opposed to acidic cellulases [10]. Neutral and alkaline cellulases have been identified and isolated from bacteria [9, 12, 13] and fungi [10, 14], and prospecting new microorganisms is constant. 

As stated above, the demand for new enzymes to develop environmentally safe processes in the textile industry will increase due to regulatory requirements adopted in most countries. Bioprospecting for new alkaline cellulase producing microorganisms from less studied environments may help to this end. Peru is a diverse country and has very broad microbial diversity richness yet little studied and exploited. Therefore, in this work Amazonian soil fungi from an undisturbed Peruvian rain forest have been isolated and tested for their ability to produce alkaline cellulases that may be used in the polishing and washing of denim processing of the textile industry.

## 2. Materials and Methods

### 2.1. Sampling Site

Soil samples were collected at five sample points from the undisturbed Macuya Forest, near Pucallpa, Peru, located at latitude 8°27′28.55′′S and longitude 74°53′44.33′′W. Average soil temperature was 24.8°C, and pH of soil samples varied between 6.5 and 7.0.

### 2.2. Soil Samples

At each 1 m^2^ sample point the leaf litter was removed and approximately 1 kg of soil was cut from the top 20 cm using a small spade and knife and divided into five 200 g samples. Samples without plant debris and stones were placed in plastic bottles and cooled for transport. Soil was homogenized manually by thorough mixing. Soil samples were then frozen at −20°C. All tools and materials used were washed and sterilized [15].

### 2.3. Primary Screening

Serial dilutions of the experimental soil samples using sterile distilled water were used for fungal isolation on agar screening medium. This medium contained, per liter, 1 g carboxymethylcellulose (CMC), 0.5 g xylose, 1 g peptone, 1 g yeast extract, 0.5 g K_2_HPO_4_, 0.5 g MgSO_4_ · 7H_2_O, 5 mg FeSO_4_ · 7H_2_O, 1.6 mg MnSO_4_ · 2H_2_O, 1.4 mg ZnSO_4_ · 7H_2_O, 2 mg CoCl_2_ · 6H_2_O, and 15 g agar. The pH of the medium was adjusted to 5.5 with either a 1 N NaOH or 1 N HCl solution. After autoclaving, oxytetracycline was added to a final concentration of 100 *μ*g mL^−1^. Inoculated plates were incubated at 28°C for five days. Selected colonies were isolated and purified on potato dextrose agar (PDA) plates and conserved on PDA slants at 4°C.

### 2.4. Secondary Screening

A semiquantitative plate clearing assay was developed based on the former method of Teather and Wood [16]. For this, the same agar screening medium without xylose and oxytetracycline was dispensed in sterile 96-well Labsystem flat-bottom ELISA microplates (LabSource, Arbor, IL, USA), each well containing 150 *μ*L agar screening medium. Small amounts of fungal spores were seeded on the top of each well and incubated at 28°C for 72 h. Four replicates were used for each fungal isolate. The content of each well (after checking the growth of the microorganism) was removed aseptically with a punch and placed on specially designed glass plates (300 cm^2^) containing the enzyme substrate solid media [17].

The enzyme substrate solid media contained, per liter buffer, 5 g CMC and 15 g agar. For pH 4.8, 7.4, 8.4, and 9.4, 0.05 mol L^−1^ citrate, phosphate buffer saline, barbital-HCl, and glycine-NaOH buffer, respectively, were used. Enzyme activity was evaluated after 4 h incubation at 50°C in a moist chamber by flooding the plates with an aqueous solution of 1 mg mL^−1^ Congo red for 15 min. The Congo red solution was then poured off, and plates were further treated by flooding with 1 mol L^−1^ NaCl for 15 min. The diameter of the clear zones was measured and taken as an indication of cellulase activity.

### 2.5. Cellulase Production in Liquid Culture

Spores of the selected strains were washed from 5-day PDA plates with 10 mL of 0.1% (v/v) Tween 80 solution, and this suspension (1 × 10^6^ spores per mL) was used as inoculum. Cellulase production medium for the growth and cellulase production had the following composition: KH_2_PO_4_ 2 g L^−1^; (NH_4_)_2_SO_4_ 1.4 g L^−1^; urea 0.3 g L^−1^; CaCl_2_ · 2H_2_O 0.3 g L^−1^; MgSO_4_ · 7H_2_O 0.3 g L^−1^; peptone 1 g L^−1^; Tween 80 0.2% (v/v); FeSO_4_ · 7H_2_O 5 mg L^−1^; MnSO_4_ · 2H_2_O 1.6 mg L^−1^; ZnSO_4_ · 7H_2_O 1.4 mg L^−1^; CoCl_2_ · 6H_2_O 2 mg L^−1^; and either lactose 10 g L^−1^ or 20 micron microcrystalline cellulose 10 g L^−1^. The pH of the medium was adjusted to 5.5 with either a 1 N NaOH or 1 N HCl solution. Each 250 mL flask containing 70 mL production medium was inoculated with a 3% (v/v) of the above spore suspension. All inoculated flasks were incubated at 28°C in a shaker bath at 175 rev min^−1^ for 72 h. Three replicates were taken in all cases. Cultures were centrifuged at 3,000 rev min^−1^ for 20 min; solids were used for biomass determination, and supernatants were used for enzyme activity and soluble protein determinations. 

### 2.6. Biomass Determination

For lactose cultures biomass was determined by measuring the dry cell weight. For cellulose cultures biomass was indirectly determined by measuring the intracellular protein contained as follows: culture solids were ground in a mortar with liquid nitrogen. Powdered biomass was gently suspended in 5 mL of extraction buffer (0.01 mol L^−1^ Tris HCL pH8; 0.001 mol L^−1^ EDTA, 2% (w/v) polyvinylpolypyrrolidone, vortexed, and centrifuged at 8000 rpm for 30 min at 4°C; then, supernatant was collected [18]. Supernatants were precipitated with methanol-chloroform following the procedure of Wessel and Fluegge [19]. Protein samples were successively mixed with methanol (4 volumes), chloroform (1 volume), and distilled water (3 volumes) and centrifuged at 14,000 rev min^−1^ at 4°C for 2 min. The upper phase was carefully removed, and 3 volumes of methanol were added followed by centrifugation at 14,000 rev min^−1^ for 2 min. The supernatant was discarded; the pellet was air-dried at room temperature and then resuspended in phosphate buffer saline. Protein concentration was estimated using the Lowry et al. [20] procedure.

### 2.7. Cellulase Activity

Cellulase, as filter paper activity (FPA), was determined according to the 96 *μ*L microplate-based (MPB) method described by Xiao et al. [21] with some modifications. Previously, standard FPA method [22] was assayed to evaluate the sensitivity and reproducibility of the MPB method. Statistical analyses showed that cellulase activities determined with the MPB method were not significantly different from the activities measured with the standard FPA (results not shown).

The MPB assay was performed as follows: a 32 *μ*L aliquot of diluted culture enzyme sample was added into UltraFlux PCR Plates (LabSource, Arbor, IL, USA) wells containing a 7 mm diameter folded filter paper disk (Whatman N° 1) and 64 *μ*L of 0.05 mol L^−1^ corresponding buffer (pH 4.8, 7.4, 8.4, or 9.4). After incubation at 50°C for 60 min, 100 *μ*L of DNS reagent was added into each plate well and incubated at 95°C for 5 min. Following color development, a 160 *μ*L aliquot of each sample was transferred to the wells of a 96-well Labsystem flat-bottom ELISA microplates (LabSource, Arbor, IL, USA) containing 85 *μ*L of distilled H_2_O and the absorbance at 540 nm was measured in a Rayto RT-2100C plate reader (Rayto Life and Analytical Sciences, Nanshan, Shenzhen, China). According to IUPAC recommendations [22], an enzyme blank (32 *μ*L aliquot of diluted enzyme and 64 *μ*L buffer) and substrate blank (96 *μ*L buffer and folded 7 mm filter paper disk) were included in each assay. One enzyme unit (U) is defined as the amount of enzyme that releases 1 *μ*mol product per minute of glucose equivalents.

### 2.8. Soluble Protein Determination

Culture supernatants were precipitated with two volumes of 10% (w/v) trichloroacetic acid, vortexed, kept overnight at 4°C, and centrifuged at 6,000 rev min^−1^ at 4°C for 25 min. Pellets were resuspended in phosphate buffer saline, and protein concentration was estimated by the Lowry et al. [20] procedure.

### 2.9. Statistics

Data were analysed by Statgraphics Centurion XVI (Statpoint Technologies, Inc., Warrenton, VA, USA). Analysis of variance (ANOVA) by the multifactor categorical statistical design was conducted in most cases. Other statistical analyses are indicated in the text, and they were also processed with the same software.

## 3. Results

### 3.1. Isolation of Fungi from Soil Samples

Five soil samples from Macuya, an undisturbed tropical rain forest (Pucallpa, Peru), were used to isolate alkaline cellulase producing fungi by using a screening medium containing CMC and xylose as carbon sources and as cellulase inducers. All soil samples show similar values of fungal propagules able to use the carbon sources (4.5–7 × 10^4^ UFC g^−1^ dry soil; Table 1). Based on differences in colony morphology, 50 colonies were isolated, purified, and subjected to secondary screening for detecting cellulase activity at different pH values. Possibly due to the design of isolation medium and short incubation time, a predominance of the genera *Aspergillus* and *Penicillium* among the 50 fungal isolates was found.

### 3.2. Screening of Cellulase Activity

The cellulolytic activity of the 50 fungal isolates was semiquantitative tested using the plate clearing assay with CMC as substrate at different pH values (see Section 2). Many fungal isolates showed cellulolytic activity even at pH 9.4. However, only those isolates that gave clear zones with diameters above 1 mm at all alkaline pH values were selected for further analysis. Therefore, 11 strains belonging to the Aspergilli and Penicilli passed the screen and were selected for cellulase production in liquid culture (Table 2). Strains *Aspergillus* sp. LM-HP32, *Aspergillus* sp. LM-HP34, *Penicillium* sp. LM-HP37, and *Penicillium* sp. LM-HP14 gave the highest clearing zones at all pH values.

### 3.3. Cellulase Production in Liquid Culture

**Figure 1:**
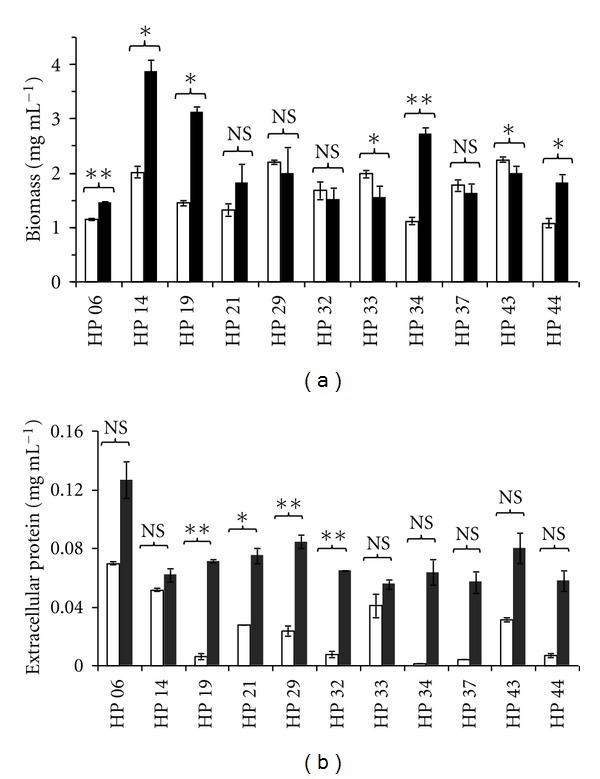
Biomass (a) and extracellular protein (b) attained on cellulose (white bars) and lactose (black bars) by eleven strains of soil fungi isolated from an undisturbed rain forest of Peru. Error bars on the graphs represent four replicates. NS: not statistically significant; *: statistically significant at 95%; **: statistically significant at 99%.

In order to determine the cellulase production capacity of the 11 selected strains, liquid cultures in shaken flasks were carried out with either microcrystalline cellulose or lactose as carbon sources for 72 h at 28°C. Growth was generally better on lactose than on cellulose for most of the strains as shown in Figure 1(a). *Penicillium* sp. LM-HP14 attained the highest biomass level in both culture media (3.85 ± 0.22 mg mL^−1^ on lactose and 2.01 ± 0.10 mg mL^−1^ on cellulose) and *Penicillium* sp. LM-HP06 the lowest (1.45 ± 0.02 mg mL^−1^ on lactose and 1.48 ± 0.39 mg mL^−1^ on cellulose). Likewise, extracellular soluble protein production was much higher on lactose in all strains (Figure 1(b)).

**Figure 2:**
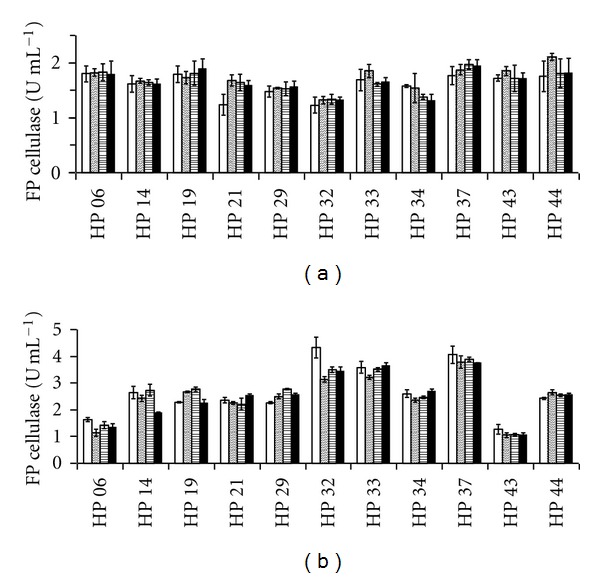
Cellulase activities attained on cellulose (a) and lactose (b) by eleven strains of soil fungi isolated from an undisturbed rain forest of Peru. Activities were measured at pH 4.8 (white bars), pH 7.4 (diagonally striped bars), pH 8.4 (horizontally striped bars), and pH 9.4 (black bars). Error bars on the graphs represent four replicates.

Filter paper cellulase activities determined at different pH values from liquid cultures on lactose and on cellulose are shown in Figure 2. Higher FP cellulase activities were produced on lactose in agreement with better growth attained on this medium. *Aspergillus* sp. LM-HP32, *Penicillium* sp. LM-HP33, and *Penicillium* sp. LM-HP37 grown on lactose were the best producers of FP cellulase (>3 U mL^−1^) at all pH values, and *Penicillium* sp. LM-HP06 and *Penicillium* sp. LM-HP43 were the worst producers (1–1.5 U mL^−1^; Figure 2(b)). However, neither strain grown on cellulose produced cellulase at levels higher than 2 U mL^−1^ at all pH values (Figure 2(a)). Interestingly, all strains produced cellulase with activities at all pH values indicating that they are true alkaline cellulase producers. Also, cellulase-specific productivities were higher on lactose (Table 3).

**Figure 3:**
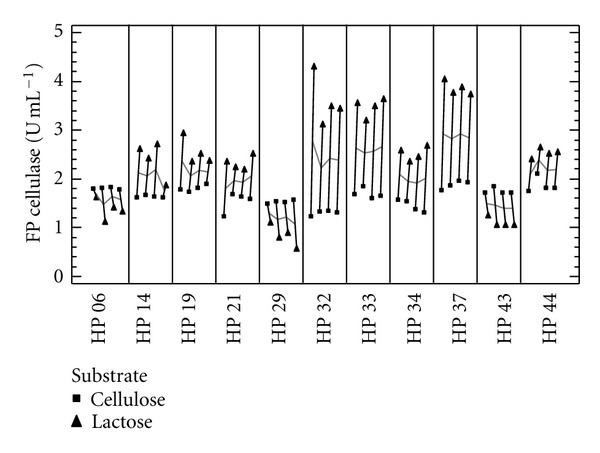
Multivari plot showing the effect of carbon source on cellulase activities at different pH values attained by eleven strains of soil fungi isolated from an undisturbed rain forest of Peru, based on a multifactor categorical statistical design. Cellulase was produced on cellulose (■) and on lactose (▲).

In order to determine the relevant factors (strain, pH, and carbon source) that influence cellulase activity, a multifactor categorical statistical design was followed. It was found that pH did not affect cellulase activity corroborating that all strains are true cellulase producers and that both strains, as expected, and carbon sources are the most important factors. Also, a multivari plot indicates that *Aspergillus* sp. LM-HP32, *Penicillium* sp. LM-HP33, and *Penicillium* sp. LM-HP37 and lactose are the best strains and substrates for alkaline cellulase production (Figure 3). 

## 4. Discussion

Fungi have many different functions in soils, which include either active roles, such as the degradation of dead plant material, or inactive roles where propagules are present in the soil as resting states. Degradation of dead plant residues is due to the activity of different types of enzymes being hydrolases the most abundant. Therefore, soils have been the preferred environments for the isolation of cellulase producing fungi [3]. Detecting exactly which fungi are present in a soil sample is no easy task; one of the major problems being the fastidious nature of the great majority of species [23]. There are few reports in international journals on soil fungal diversity of Peru [24], and this is particularly the worst for the Amazonian rain forest of this country. Given the high carbon turnover of rain forest soils and the characteristics of these environments, it was assumed that cellulase producing soil fungi would be highly diverse having enzymes with interesting activities. Therefore, the undisturbed Macuya rain forest (Pucallpa, Peru) was selected for the isolation of fungi producing alkaline cellulases, and, as far as it is known, this is the first paper on the topic.

The soil dilution plate method was used for the enumeration and isolation of fast growing cellulolytic fungi on an enriched selective medium. Numbers of colony forming units per gram of dry soil as presented in Table 1 are higher than those reported elsewhere for saprophytic fungi in different types of soils, indicating high degrading activity of plant biomass [25–27]. Due to the selection procedure final selected strains producing high alkaline cellulase levels were members of the Penicilli and Aspergilli groups since they are fast growing and highly sporulating fungi. Representatives of these fungal groups with alkalophilic or thermophilic enzymes are currently isolated from soils [28–30].

Out of fifty different fungal isolates that grew on the CMC-xylose isolation medium, 11 were finally selected by using the plate clearing assay with CMC as substrate at different pH values (Table 2). Although semiquantitative, this assay gives a fairly good correlation with production assays in liquid cultures and it is, therefore, useful for the rapid screening of large numbers of fungal colonies under various conditions [16, 31]. All of the 11 strains produced cellulases with activities at alkaline pH values without an apparent decrease of them indicating that they are true alkaline cellulase producers. However, many of them had better enzyme activities when grown on lactose as shown in Figure 2, although their growth was not necessarily better. This may be due to the short incubation time (72 h) employed in this experiment as growth on cellulose requires more time for its total consumption. Also, lactose is frequently used as a good cellulase inducer in several fungal species [32–34]. Multivari plots are useful for deciding the best strains when comparing various influencing factors (Figure 3). In this sense, three strains (*Aspergillus* sp. LM-HP32, *Penicillium* sp. LM-HP33, and *Penicillium* sp. LM-HP37) have shown to be suitable for alkaline cellulase production under industrial conditions as they also have higher specific productivities, an important industrial parameter (Table 3).

In conclusion, the three strains selected in this work may be further studied to develop processes for alkaline cellulase production that is demanded by the modern textile industry. Soils from Amazonian rain forests are good sources of industrial fungi with particular characteristics. Soil fungal diversity of these soils has been barely studied and deserves more attention. We are conducting bioprospecting work searching for cell-wall degrading enzyme genes from this environment.

## Figures and Tables

**Table 1:** Number of viable fungi counts from each soil sample.

Soil	CFU g^−1∗^	First selected isolates^†^	Genera (number of colonies)	Final selected isolates^‡^
1	4.5 × 10^4^	13	*Aspergillus* (1), *Byssochlamys* (1), *Fusarium* (1), *Penicillium* (5), *Trichoderma* (2), unknown (3)	3

2	3.5 × 10^4^	8	*Paecilomyces* (1), *Aspergillus* (4), *Cladosporium* (1), *Mucor* (2), *Penicillium* (2), unknown (7)	2

3	6.5 × 10^4^	17	*Acremonium* (1), *Penicillium* (2), *Trichoderma* (1), unknown (4)	6

4	7.0 × 10^4^	6	*Penicillium* (3), unknown (3)	0

5	6.5 × 10^4^	6	*Penicillium* (1), unknown (5)	0

*Colony-forming units per gram of dry soil.

^†^Number of different strains isolated in pure culture based on colony morphology.

^‡^Number of fungal isolates that passed the semiquantitative screening at all pH values tested.

**Table 2:** Semiquantitative endoglucanase activity of selected fungal isolates from Macuya forest soils (Pucallpa, Peru).

Fungal isolates	Endoglucanase activity expressed as clear zones (mm)			
	pH 4.8	pH 7.4	pH 8.4	pH 9.4
*Penicillium* sp. LM-HP06	1.2 ± 0.07	1.1 ± 0.07	1.2 ± 0.07	1.3 ± 0.00
*Penicillium* sp. LM-HP14	1.4 ± 0.00	1.2 ± 0.04	1.4 ± 0.04	1.3 ± 0.14
*Penicillium* sp. LM-HP19	1.2 ± 0.04	0.9 ± 0.07	1.2 ± 0.00	1.1 ± 0.04
*Penicillium* sp. LM-HP21	1.3 ± 0.00	1.0 ± 0.00	0.9 ± 0.00	1.4 ± 0.04
*Aspergillus* sp. LM-HP29	1.0 ± 0.00	1.0 ± 0.00	1.1 ± 0.14	1.1 ± 0.07
*Aspergillus* sp. LM-HP32	1.4 ± 0.00	1.4 ± 0.00	1.5 ± 0.00	1.5 ± 0.00
*Penicillium* sp. LM-HP33	1.2 ± 0.07	1.1 ± 0.00	1.2 ± 0.00	1.3 ± 0.07
*Aspergillus* sp. LM-HP34	1.3 ± 0.07	1.4 ± 0.00	1.6 ± 0.04	1.5 ± 0.00
*Penicillium* sp. LM-HP37	1.3 ± 0.00	1.3 ± 0.00	1.3 ± 0.00	1.4 ± 0.07
*Penicillium* sp. LM-HP43	1.0 ± 0.00	1.2 ± 0.00	1.4 ± 0.00	1.4 ± 0.00
*Aspergillus* sp. LM-HP44	0.0 ± 0.00	1.0 ± 0.00	1.3 ± 0.00	1.3 ± 0.0

Values represent mean of four replicates ± SD.

**Table 3:** Cellulase-specific productivities on lactose.

Strain	FP cellulose-specific productivity (U g^−1^ h^−1^)			
	pH 4.8	pH 7.4	pH 8.4	pH 9.4
*Penicillium* sp. LM-HP06	15.6 ± 0.9	10.8 ± 1.3	13.7 ± 1.3	12.9 ± 1.1
*Penicillium* sp. LM-HP14	9.5 ± 0.9	8.7 ± 0.8	9.8 ± 1.2	6.8 ± 0.4
*Penicillium* sp. LM-HP19	10.1 ± 0.3	11.8 ± 0.3	12.3 ± 0.5	10.0 ± 0.6
*Penicillium* sp. LM-HP21	18.5 ± 3.0	17.8 ± 3.6	17.5 ± 4.3	19.9 ± 3.9
*Aspergillus* sp. LM-HP29	16.5 ± 4.0	18.3 ± 4.5	20.2 ± 4.9	18.6 ± 4.5
*Aspergillus* sp. LM-HP32	40.9 ± 9.4	29.3 ± 3.5	32.8 ± 4.7	32.2 ± 3.2
*Penicillium* sp. LM-HP33	32.9 ± 6.0	29.4 ± 3.9	32.2 ± 5.0	33.6 ± 5.6
*Aspergillus* sp. LM-HP34	13.3 ± 0.3	12.1 ± 0.5	12.6 ± 0.5	13.8 ± 0.5
*Penicillium* sp. LM-HP37	34.7 ± 4.5	32.3 ± 3.8	33.3 ± 3.5	32.1 ± 3.3
*Penicillium* sp. LM-HP43	9.0 ± 1.6	7.4 ± 0.6	7.5 ± 0.6	7.5 ± 1.0
*Aspergillus* sp. LM-HP44	18.6 ± 1.6	20.5 ± 2.5	19.5 ± 1.5	19.7 ± 1.6

Values represent mean of four replicates ± SD.
